# Supplementary material for: Loss of genetic variation and ancestral sex determination system in North American northern pike characterized by whole-genome resequencing
Source: G3 (Bethesda). 2024 Aug 8;14(10):jkae183. doi: 10.1093/g3journal/jkae183 (PMC11457062; doi:10.1093/g3journal/jkae183)
Supplement: jkae183_Supplementary_Data [file jkae183_supplementary_data.zip › Supplemental_Material_Legends_G3-2024-405269.docx]

**Supplemental Material Legends**

**File S1.** Hi-C contact map as visualized in Juicebox v 1.8.8 for assembly v4.0 GCF_004634155.1.

**File S2.** Distribution of repeats along the assembly v4.0 GCF_004634155.1, displayed in bins of 100,000 bp along the chromosome, with counts of masked bases per bin.

**File S3.** Counts and locus identifiers of heterozygous or homozygous alternate variants per individual for all individuals in the study.

**File S4.** Sum of variants per window across the genome in 1 Mbp windows.

**File S5.** Sex-specific SNPs identified from the regional analysis of the whole-genome sequence data, shown per genomic coordinate as a 0 (ref/ref) or 1 (ref/alt) for each sexed northern pike from Chatanika River.

**Table S1.** Primer names, sequences, and annealing temperatures for sex markers. SeqAMH1 and ConserveAMH1 are reported in Pan et al. (2021).

**Table S2.** RepeatMasker summary table for assembly version 4.0 (GCF_004634155.1). Description of custom repeat library generation and masking methods described in Rondeau et al, (2014).

**Table S3.** Coverage for select individuals from across the sampled range on *amhby* and LG01, and relative depth of coverage. The *amhby* scaffold originates from from Pan et al. (2019) and was added to the v.4.0 reference genome for these alignments only.
