# Supplementary material for: Loss of genetic variation and ancestral sex determination system in North American northern pike characterized by whole-genome resequencing
Source: G3 (Bethesda). 2024 Aug 8;14(10):jkae183. doi: 10.1093/g3journal/jkae183 (PMC11457062; doi:10.1093/g3journal/jkae183)
Supplement: jkae183_Supplementary_Data [file jkae183_supplementary_data.zip › Supplemental_Tables_G3-2024-405269.docx]

**Supplemental Information for:**

**Loss of genetic variation and ancestral sex determination system in North American northern pike characterized by whole-genome resequencing**

Hollie A. Johnson, Eric B. Rondeau, Ben J. G. Sutherland, David R. Minkley, Jong S. Leong, Joanne Whitehead, Cody A. Despins, Brent E. Gowen, Brian J. Collyard, Christopher M. Whipps, John M. Farrell, Ben F. Koop

**Table of Contents:**

| **Table S1. Primer information** | **Page 2** |
| --- | --- |
| **Table S2. Repeats summary** | **Page 3** |
| **Table S3. Coverage against *amhby*** | **Page 4** |

**Table S1.** Primer names, sequences, and annealing temperatures for sex markers. SeqAMH1 and ConserveAMH1 are reported in Pan et al. (2021).

| **Primer name** | **Sequence 5' → 3'** | **Annealing temp. (˚C)** |
| --- | --- | --- |
| 24.5 F | AATTACAGACCTCTACATGCT | 52 |
| 24.5 R | GATAGTCCCATAGATTGTAGA |  |
| 24.5 Probe | GCAAATGACGGCGCACTGTT |  |
| SeqAMH1Fw4 | CAACATGGTGGCAACTAAGTG | 52 |
| SeqAMH1Rev4 | GGTAATATTTGTGCCCTGTG |  |
| ConserveAMH1_F1 | GTTACTTTTTCTGCCTAGCGTGA | 54 |
| ConserveAMH1_R1 | CTATTACTAGTGTGGATAAGGCCG |  |

**Table S2.** RepeatMasker summary table for assembly version 4.0 (GCF_004634155.1). Description of custom repeat library generation and masking methods described in Rondeau et al. (2014).

|  | **GC level:** | 42.29% |  |
| --- | --- | --- | --- |
|  | **Bases masked:** | 393,803,600 bp (41.85%) |  |
|  | **Number of elements** | **Length occupied (bp)** | **Percentage of sequence** |
| **SINEs:** | 18,129 | 1,946,749 | 0.21% |
| **ALUs** | 0 | 0 | 0.00% |
| **MIRs** | 0 | 0 | 0.00% |
| **LINEs:** | 132,531 | 61,080,041 | 6.49% |
| **LINE1** | 4,112 | 1,212,654 | 0.13% |
| **LINE2** | 52,751 | 23,286,100 | 2.47% |
| **L3/CR1** | 620 | 289,097 | 0.03% |
| **LTR elements:** | 35,971 | 23,113,357 | 2.46% |
| **ERVL** | 0 | 0 | 0.00% |
| **ERVL-MaLRs** | 0 | 0 | 0.00% |
| **ERV_classI** | 0 | 0 | 0.00% |
| **ERV_classII** | 0 | 0 | 0.00% |
| **DNA elements:** | 556,653 | 190,993,033 | 20.30% |
| **hAT-Charlie** | 0 | 0 | 0.00% |
| **TcMar-Tigger** | 0 | 0 | 0.00% |
| **Unclassified:** | 482,717 | 96,062,470 | 10.21% |
| **Total Interspersed Repeats:** | *NA* | 373,195,650 | 39.66% |
| **Small RNA:** | 13,404 | 1,418,045 | 0.15% |
| **Satellites:** | 6,505 | 2,404,400 | 0.26% |
| **Simple repeats:** | 264,557 | 14,870,677 | 1.58% |
| **Low complexity:** | 38,843 | 2,883,801 | 0.31% |

**Table S3.** Coverage for select individuals from across the sampled range on *amhby* and LG01, and relative depth of coverage. The *amhby* scaffold originates from from Pan et al. (2019) and was added to the v.4.0 reference genome for these alignments only.

| **Location** | **Abbr.** | **Individual** | **Sex** | ***amhby* mean coverage** | **LG01 mean coverage** | **Relative coverage (amhby/LG01)** |
| --- | --- | --- | --- | --- | --- | --- |
| Chatanika R | CHT | CR1 | F | 1.78 | 31.4 | 0.06 |
| Chatanika R | CHT | CR2 | F | 1.42 | 27.3 | 0.05 |
| Chatanika R | CHT | CR3 | F | 1.36 | 27.9 | 0.05 |
| Chatanika R | CHT | CR4 | F | 1.18 | 28.3 | 0.04 |
| Chatanika R | CHT | CR6 | F | 1.47 | 27.1 | 0.05 |
| Chatanika R | CHT | CR5 | M | 30.06 | 38.0 | 0.79 |
| Chatanika R | CHT | CR7 | M | 27.72 | 33.0 | 0.84 |
| Chatanika R | CHT | CR8 | M | 21.44 | 26.6 | 0.81 |
| Chatanika R | CHT | CR9 | M | 21.7 | 27.2 | 0.80 |
| Chatanika R | CHT | CR10 | M | 21.97 | 27.1 | 0.81 |
| Yukon R | HOO | YR1 | U | 18.5 | 21.4 | 0.86 |
| Yukon R | HOO | YR2 | U | 0.57 | 20.4 | 0.03 |
| Palmer Lake | PAL | PL2 | U | 0.62 | 19.8 | 0.03 |
| Palmer Lake | PAL | PL3 | U | 0.66 | 22.7 | 0.03 |
| Palmer Lake | PAL | PL4 | U | 0.5 | 20.2 | 0.02 |
| Whiteshell | WHI | Mb7 | F | 0.58 | 22.4 | 0.03 |
| Whiteshell | WHI | Mb8 | M | 0.52 | 19.2 | 0.03 |
| St. Lawrence | SLA | S20 | F | 0.53 | 20.3 | 0.03 |
| St. Lawrence | SLA | S25 | M | 0.64 | 22.7 | 0.03 |
| Hackettstown | HCK | NJ5 | F | 0.95 | 21.5 | 0.04 |
| Hackettstown | HCK | NJ10 | M | 0.65 | 22.4 | 0.03 |
