# Supplementary figures and images for: Loss of genetic variation and ancestral sex determination system in North American northern pike characterized by whole-genome resequencing
Source: G3 (Bethesda). 2024 Aug 8;14(10):jkae183. doi: 10.1093/g3journal/jkae183 (PMC11457062; doi:10.1093/g3journal/jkae183)

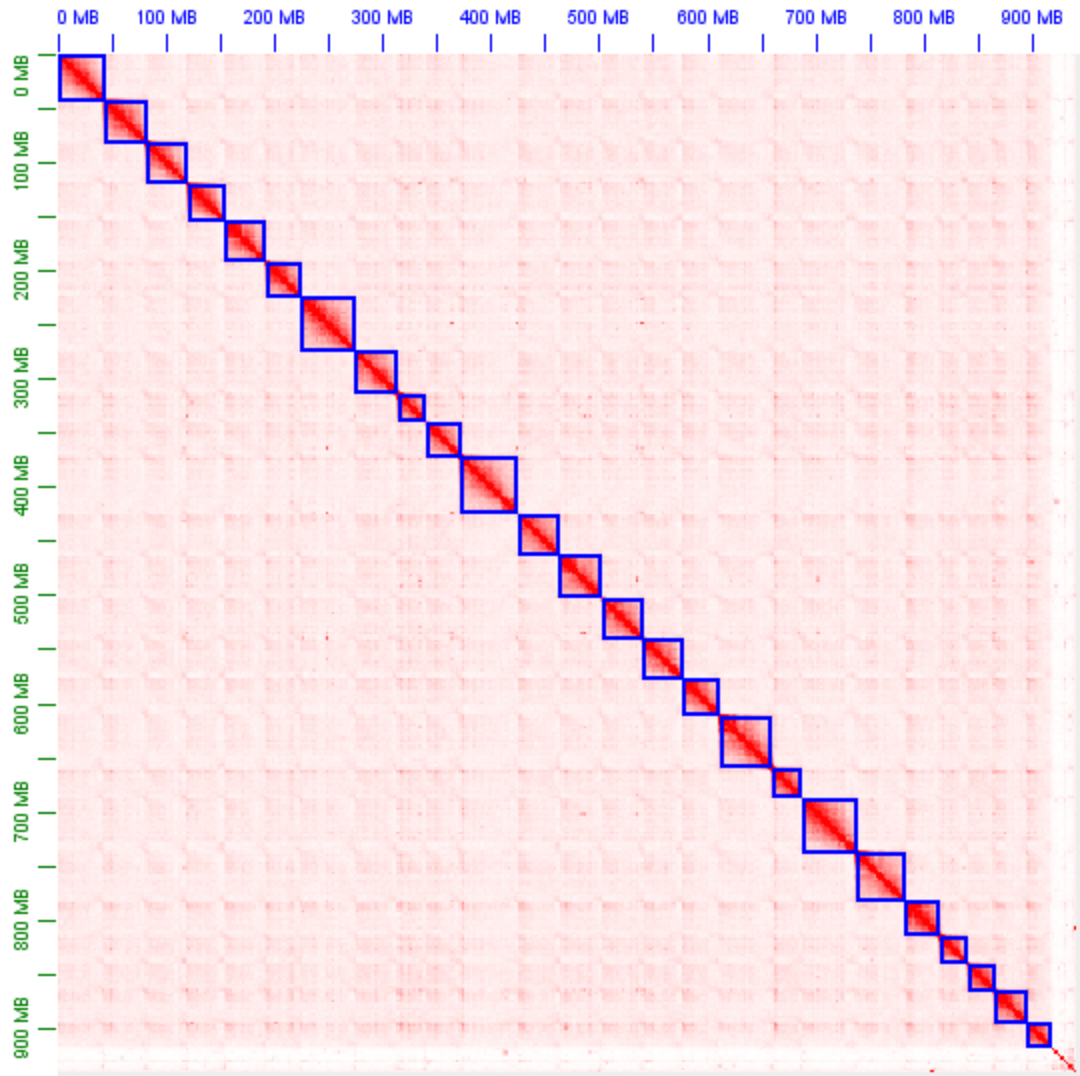

Supplement: jkae183_Supplementary_Data [file jkae183_supplementary_data.zip › File_S1_G3-2024-405269.pdf]

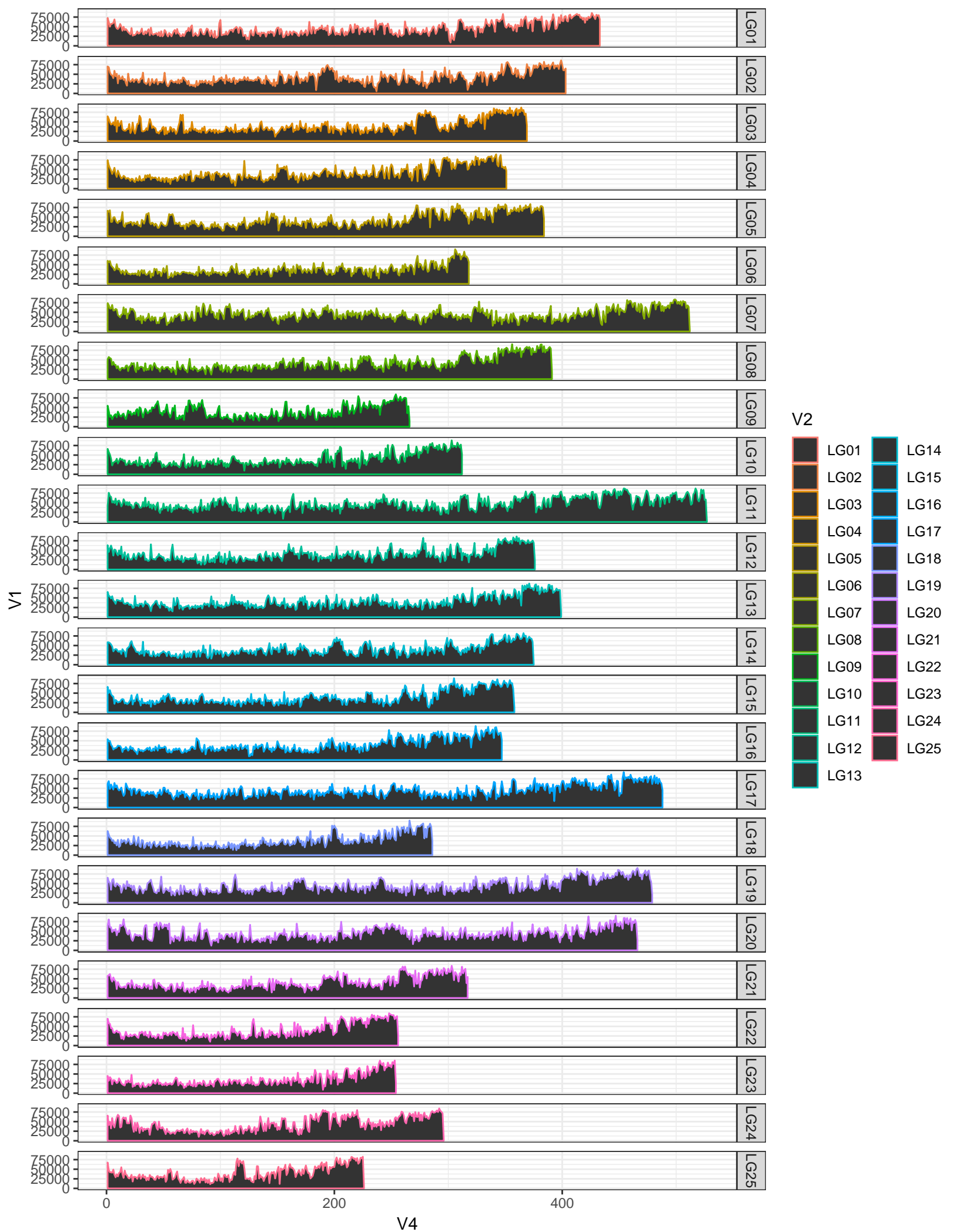

Supplement: jkae183_Supplementary_Data [file jkae183_supplementary_data.zip › File_S2_G3-2024-405269.pdf]
